# Methods for questionnaire design: a taxonomy linking procedures to test goals

**DOI:** 10.1007/s11136-019-02209-6

**Published:** 2019-05-18

**Authors:** Paul Oosterveld, Harrie C. M. Vorst, Niels Smits

**Affiliations:** 1 Developmental Psychology, Leiden University, Leiden, The Netherlands; 2 Department of Psychology, University of Amsterdam, Amsterdam, The Netherlands; 3 Research Institute of Child Development and Education, University of Amsterdam, Nieuwe Achtergracht 127, 1018 WS Amsterdam, The Netherlands

**Keywords:** Test construction, Questionnaire design, Validity, Measurement

## Abstract

**Background:**

In the clinical field, the use of questionnaires is ubiquitous, and many different methods for constructing them are available. The reason for using a specific method is usually lacking, and a generally accepted classification of methods is not yet available. To guide test developers and users, this article presents a taxonomy for methods of questionnaire design which links the methods to the goal of a test.

**Methods:**

The taxonomy assumes that construction methods are directed towards psychometric aspects. Four stages of test construction are distinguished to describe methods: concept analysis, item production, scale construction, and evaluation; the scale construction stage is used for identifying methods. It distinguishes six different methods: the rational method utilizes expert judgments to ensure face validity. The prototypical method uses prototypicality judgments to ensure process validity. In the internal method, item sets are selected that optimize homogeneity. The external method optimizes criterion validity by selecting items that best predict an external criterion. Under the construct method theoretical considerations are used to optimize construct validity. The facet method is aimed at optimizing content validity through a complete representation of the concept domain.

**Conclusion:**

The taxonomy is comprehensive, constitutes a useful tool for describing procedures used in questionnaire design, and allows for setting up a test construction plan in which the priorities among psychometric aspects are made explicit.

## Introduction

The use of tests and questionnaires in the behavioral sciences and psychiatry can be dated back as far as a century ago with the development of Woodworth’s Personal Data Sheet [1] and has become widespread. Likewise, in the relatively young field of (health-related) quality of life research, questionnaires also play a central role. In the last decades, the construction of questionnaires has therefore become a highly relevant and vital activity; to illustrate, a quick search on Google Scholar using the term “test construction” gave more than 50,000 hits. A questionnaire is defined, here, as an instrument for the measurement of one or more constructs by means of aggregated item scores, called scales. The items of a questionnaire are usually completely structured: they have a similar format, are usually statements, questions, or stimulus words with structured response categories, and require a judgment or description by a respondent or rater. A method of questionnaire construction refers to the procedure followed in constructing a measurement instrument. Information about the construction of questionnaires can be found in scientific journals (e.g., [2–5]), text books on assessment and testing (e.g., [1, 6–11]), standards for psychological testing [12], standards for measuring quality of life [13, 14], guidance for medical product development [15], manuals of questionnaires (e.g., [16–18]), and documented reviews of questionnaires and tests for practitioners (e.g., [19]). All these sources are characterized by relatively little attention to the construction of the questionnaire. Their emphasis is instead on requirements for a questionnaire (e.g., a full coverage of an affective domain). The specific procedures (to be) followed (e.g., the use of a facet design) often remain unmentioned and the choice for a specific procedure is not often substantiated.

A multitude of methods for constructing questionnaires exist [20, 21], and there have been attempts to arrange them into classes [20, 22–31], but these have not resulted in a generally accepted taxonomy. There may be several reasons for this. Possibly, the lack of consensus in the terminology used [20]; for example, the term ‘rational method’ is used by several authors to refer to radically different procedures [25, 26, 31, 32]. Similarly, there are large differences in abstraction level used to classify the methods. The so-called ‘deductive’ and ‘inductive’ methods of Burisch [23], for example, are presented as broad categories, whereas the methods of the same name discussed by Hermans [33] refer to quite specific methods of item selection. Another reason may be that previous classifications of methods have not been comprehensive; for example, the method based on the evaluation of prototypicality of items ([34], see below) has not appeared in overviews of methods of questionnaire design. Most important, although the available classifications divide the procedures according to their similarity in steps and actions taken, they fail to demonstrate *why* these procedures are chosen.

In the current article, we propose a new taxonomy for methods of test construction that links the methods to the goal of a test construction. More specifically, it distinguishes six types of procedures that each relate to a different psychometric aspect of questionnaires. The remainder of this article is broken into three main sections. First, the general structure of the taxonomy is introduced. Next, a detailed description of each of the six methods is presented, and the article closes with a discussion of the usefulness of the taxonomy and its relation with current validity theory.

## A taxonomy of questionnaire design methods

**Table 1:** Description of the six questionnaire design methods using four stages of test construction

	Class					
	Intuitive		Inductive		Deductive	
Method Aspect	Rational Face validity	Prototypical Process validity	Internal Homogeneity	External Criterion validity	Construct Construct validity	Facet Content validity
Concept analysis	Working definition	–	–	–	Nomological network, precise definitions	Facets and facet elements
Item production	Informal criteria	Act nomination	Homogeneous	Heterogeneous	Based on definitions	Based on mapping sentence
Scale construction	**Face validity**	**Prototypicality ratings**	**Homogeneity analysis**	**Item-criterion relation**	**Convergent and discriminant item validities**	**Dimensionality analysis**
Evaluation	Diagnostic comparison	Reliability, validity	Cross-validation, test post hoc theory	Cross-validation, retest reliability	Reliability, convergent, and discriminant validity	Cross-validation, reliability, validity

The first two rows present three broader classes of design methods. Below each design method, its target psychometric aspect is provided. The content of the scale construction stage is bold-faced for each method to emphasize that the taxonomy uses this stage for classification

The taxonomy is introduced using Table 1, in which the columns contain the six questionnaire design methods: The rational, prototypical, internal, external, construct, and facet design method. These methods are the result of a literature review described elsewhere [20, 35], and are related to six psychometric features guiding them (see, third row of Table 1): face validity, process validity (a feature which is introduced later), homogeneity, criterion validity, construct validity, and content validity.

The methods are described using the four stages that are typically encountered in questionnaire construction (see, the rows of Table 1): concept analysis, item production, scale construction, and evaluation (cf. [8, 36, 37]). The *concept analysis* is the definitional stage in which the theoretical framework is identified and definitions of the constructs are made. In the *item production* stage, an item pool is produced or obtained, based on specifications made in the concept analysis. This phase can also comprise an item review by judges, e.g., experts, or potential respondents, and a pilot administration of the preliminary questionnaire, the results of which are subsequently used for refinement of the items. In the *scale construction* stage, items are selected for the scales based on a selection procedure that optimizes the psychometric aspect central in the method. In the *evaluation* phase, both the central and other relevant psychometric aspects of the final form of the questionnaire are evaluated. In this outline, one stage seemingly leads to the next, but in practice the construction of questionnaires is complex and has an iterative nature. For example, in the item production stage it may turn out that the concept analysis was incomplete and one has to take a step back to make appropriate adjustments. In addition, this outline leaves out some steps that are often taken, such test norming, because they are similar for all methods and because commonly they are inconsequential for the content of the questionnaire. Furthermore, for three methods, the prototypical, internal, and external, the cells in the table associated with the concept analysis are left blank because this stage either cannot be classified by a single framework, or is very limited in content.

Although the taxonomy uses all four stages to describe procedures followed in questionnaire design, the scale construction stage determines to which of the six classes a procedure is assigned. In this stage, it is decided what psychometric aspect is given priority to when selecting items into a scale, which is decisive for the characteristics of a questionnaire, and therefore it is considered of paramount importance in the taxonomy.

The six methods may be further clustered into three more general classes of methods, based on the type of procedure that is used to ensure the validity of the novel questionnaire (see, the first and second row of the table). Both the rational and the prototypical method use personal evaluations, by which they have an *intuitive* basis. The internal and external method seek validity through the use of empirical data, the former focusing on observed relations among items, and the latter on the relationships between items and an external criterion. Because such relationships emerge from the data, these methods are labeled *inductive*. The construct and facet method are based on a conceptual and theoretical framework, respectively, and because these methods are guided by testing hypotheses, they are labeled *deductive*.

Because of its teleological nature, the presented taxonomy has a similar philosophy as the way in which Cook and Campbell [38, 39] evaluated experimental and quasi-experimental research, linking the appropriateness of designs and methods to the purpose of a study. For example, randomized experiments and regression discontinuity analysis are appropriate when a research study is mainly concerned with causal inference. In addition, Cook and Campbell emphasized that studies cannot comply with all methodological requirements, and thus that trade-offs may exist. For example, in studies that use methods that allow for answering causal questions it is often hard to generalize findings to other populations and settings; conversely, studies that allow for generalizing findings are often less suitable to make causal claims [39]. In a similar fashion, our taxonomy specifies that each method is directed towards a specific psychometric aspect of a questionnaire, and that due to the existence of trade-offs, optimizing one aspect of a questionnaire may cause its other aspects to be suboptimal. This means that if a test constructor mostly values, and therefore optimizes, one aspect, the resulting questionnaire may not perform as well on an alternative aspect as when that aspect had been valued mostly and an appropriate method had been used for item selection. Note that this does not preclude the construction of a questionnaire that does well on multiple aspects. A questionnaire may meet the minimal requirements for several psychometric aspects, but it is unlikely that it is *optimal* for each of those.

The current taxonomy has two goals. The first goal is to provide an instructive tool to assist both developers of questionnaires and students learning about test construction. It may be used to distinguish between the different psychometric aspects and to pinpoint the differences and similarities between questionnaires and their construction methods. A second goal is to inform scholars in the field of quality of life of the variety of questionnaire design methods within their and related fields such as psychology and psychiatry.

## The six questionnaire design methods

### The rational method

In the rational method, which is guided by face validity, the knowledge of experts plays a crucial role [27, 30]. The empirical underpinnings of this knowledge is not of great concern, and the method is appropriate when the constructs of interest have been explored only superficially or when little formal knowledge is available. The term ‘rational’ refers to the supposed rationality of the considerations of experts [27]. It is the oldest method known [40], and has also been referred to as the ‘intuitive’ [33, 41], ‘pre-theoretical’ [30], and the ‘non-theoretical’ method [26]. Examples of questionnaires constructed using the rational method are the Parental Beliefs about Anxiety Questionnaire [42] and the Peritraumatic Behavior Questionnaire [43].

The theoretical framework used in the *concept analysis* is generally provided by the developer’s ideas about the construct. These ideas, usually expressed in a working definition, are implicit hypotheses based on formal or informal observations, empirical results, or a review of the literature. The construct is often specified in typologies, syndromes, or global descriptions, and the working definition is usually elaborated using the knowledge of experts (clinicians, teachers, managers, etc.) or respondents.

In its *item production* stage, the rational method uses intuitive or informal criteria. Often items are produced using the available typologies, syndromes, and global descriptions. The material collected by means of interviews with experts, essays, clinical cases, etc., may also provide suggestions for item content. An item review procedure may be incorporated as well to assure face validity. For example, experts or patients are asked to judge the items in the initial pool [44]. If feasible, poor items are rewritten, otherwise they are discarded.

The *scale construction* is based on the experts’ or constructor’s judgment. In this step, each item is assessed with respect to its face validity for measuring the construct. Usually, the assessment is carried out by a team and the decision to exclude an item is based on a vote. In addition, the experts may provide cut-off scores or interpretative categories (e.g., diagnostic criteria) for item selection.

The *evaluation* is usually rather concise because the experts’ judgment of the items are supposed to ensure the relevance of the items and the face validity of the instrument (e.g., [45]). Sometimes, comparisons are carried out between results based on the questionnaire and results based on a clinical evaluation. Also, sometimes other psychometric criteria such as estimates of reliability and validity are evaluated, but there is there is no guarantee that the scale performs well.

### The prototypical method

The prototypical method [32], also known as the ‘act frequency approach’ [46, 47], is based on prototype theory, a theory from cognitive science about the representation of categories [48, 49]. According to this theory, members of a category vary in the extent to which they are characteristic of the category; the member most characteristic, i.e., prototypical, of the category, is easiest to categorize. Applied to test construction, constructs are represented by sets of behavior (called acts), and some acts are considered to be more prototypical of the construct than others. By focusing on items that are related to prototypical acts, the respondent’s cognitive representation of the construct and the item content are assumed to coincide by which the quality of the questionnaire is ensured. As the prototypical method focuses on the cognitive process of stimulus representation, the term ‘process validity’ [50] is used to denote the aspect guiding this method [51]. Construction according to the prototypical method is guided by the (informal) knowledge and experience of the respondents. It has been recommended for the specification of implicit ideas and operationalization of concepts that are difficult to define [52]. Examples of questionnaires designed according to the prototypical method are the Social Generativity Scale [53] and the Behavioral Indicators of Conscientiousness [54].

Commonly, a *concept analysis* is absent and construction starts with the production of the items (see the blank cell for this stage in Table 1). Even if available, formal theory concerning the construct is not used because it provides no information about the prototypical structure of the construct [52].

The *item production* is based on the so-called act nomination: a sample of members from the target population is instructed to think of persons with extreme positions on the construct to be operationalized, and to write down behaviors that exemplify this construct. To ensure the prototypicality of this preliminary set of items, editing by the developer is kept to a minimum.

In the *scale construction* stage, prototypicality ratings are used for selecting items. Usually, a new sample from the target population is taken, and the participants rate the prototypicality of each item on Likert type response scales. The higher the ratings, the higher the assumed quality of the item; items with high mean ratings are included in the scale.

In the *evaluation* stage, the prototypicality principle itself is not used because the prototypicality of the items, and process validity of the scales, are assumed to have been accomplished by the act of nomination and prototypicality rating procedures. However, this stage often consists of a peer-rating procedure [52, 55]. Frequently, other criteria, such as reliability and dimensionality, are evaluated, but it cannot be known in advance how well the scales perform.

Like the rational method, the prototypical method belongs to the class of intuitive methods. Both methods have in common that the inclusion of items into a scale is based on the evaluations of one or more persons. The most apparent difference is that the evaluation stage of the prototypical method is more systematic and extensive, using standardized evaluations and a large sample of judges.

### The internal method

The internal method is guided by the assumption that constructs cannot be specified in advance, but must be derived from empirical relations between items (cf. [56–58]). In this method, it is assumed that the observed covariance among a set of items is attributable to a common factor, which is interpreted as the underlying construct. The meaning of the items and the number of scales and constructs are based on the structure of the data. The method is often used to improve an existing instrument, or to construct a new instrument from a collection of questionnaires sharing a domain, and is also known as the ‘inductive’ [23], and ‘factor analytic’ method [26]. Examples of questionnaires constructed according to the internal method are the 16 Personality Factors Questionnaire [56] and the Revised NEO Personality Inventory [59]. Using this method, the PROMIS initiative [60] has produced a large number of item collections (called ‘item banks’) for various constructs relevant for assessing quality of life in the medical field, such as physical functioning, fatigue, and pain.

The internal method typically contains no *concept analysis* because constructs are derived from the data (see the blank cell for this stage in Table 1). If it is encountered, it is usually rather modest, such as a rough specification of the content domain (e.g., ‘health-related quality of life’ or ‘personality’). Questionnaire construction typically starts with the production of the items.

In the *item production* stage, the main requirement is that the items are relevant for the content domain, and as a consequence, that they show some degree of content homogeneity. Although the internal method does not preclude producing new items (cf. [57]), it is often found that this stage consists of selecting existing sets of items, such as when combining the items of several questionnaires with a similar content domain [60, 61].

In the *scale construction* stage the internal method focuses on the homogeneity of items. Many techniques are available for obtaining homogeneous scales. Classical methods include item-rest correlations and Cronbach’s alpha, exploratory factor analytic, and componential procedures^1^ [57, 61, 63–66]. Modern methods include item response theory (e.g., [67]) and confirmatory factor analysis (e.g., [68]). The sets of items that are identified as homogeneous are interpreted post hoc, and the meaning of a given scale is derived from the content of its constituting items. Items that fail to show homogeneity are typically removed.

In the *evaluation* stage, the stability of the identified inter-item covariance structure is usually assessed. To that end, the established model is fit in a new sample of respondents from the target population, and this stage is therefore characterized by the use of both confirmatory techniques, and cross-validation [69]. If the inter-item structure does not change much, it is expected that the scales perform well on measures associated with homogeneity. By contrast, the failure to cross-validate is usually interpreted as a misspecification of the original model, possibly due to capitalization on chance (a.k.a. ‘overfitting’ [70, 71]); the interpretation of the new covariance structure guides adjustments of the original scale. Because the internal method focuses on empirical relationships among the items, it cannot be known in advance if the resulting scale performs well on other criteria such as face validity and the predictive validity of an external criterion (see, next section).

### The external method

The external method is guided by the empirical relationship of the questionnaire with an external criterion. This relationship comes in two major forms: concurrent and predictive [72]. The former concerns the association with a criterion obtained at the same point in time, whereas the latter with a criterion obtained in the future. Orthogonal to this distinction is the reason for the focus on this relationship ([73, Chap. 10]). First, it is used as a proof of the questionnaire measuring a theorized construct: if this construct is expected to be related to the criterion, an empirical relation between the questionnaire and the criterion may be seen as proof of its validity. Second, to ensure the utility of the questionnaire for predicting the criterion. The criterion usually is a variable that is theoretically or practically relevant, such as a behavioral measure (e.g., utilization of medical services), judgments by others (e.g., peer- or parent ratings), group membership (e.g., vocational group), or clinical status (e.g., ‘diseased’ versus ‘healthy’).

The external method gained popularity in the 1950s when behaviorism dominated psychology, and it was thought that responses to questionnaire items are in themselves interesting pieces of behavior, that may be related to non-test behavior [74, 75]. In addition, the method has also been used in two-stage testing in which a questionnaire, often referred to as ‘screener,’ serves as a first test (e.g., [76, 77]). The second stage consists of an extensive (i.e., expensive) examination of the individual, often referred to as the gold standard. The external method is also known as ‘criterion-keying,’ the ‘criterion oriented’ [31], the ‘empirical’ [26], and the ‘actuarial’ method [7]. Well-known questionnaires developed by means of this method are the Minnesota Multiphasic Personality Inventory [16], and the California Psychological Inventory [78]. In addition, many screeners for detecting patients with high risk of pathology have been constructed using this method (also, see [77]); examples are the Patient Health Questionnaire-Depression [79], and the Generalized Anxiety Disorder Assessment [80].

The *concept analysis* stage of the external method is typically very modest in size or absent (see the blank cell for this stage in Table 1), because the content of the questionnaire is determined by the criterion variable, and not by a theoretical construct.

In the *item production* stage, a collection of heterogeneous items that seem relevant for the criterion is obtained [81]; hence, the item set typically touches on many different aspects of the construct. Although sometimes new items are constructed (e.g., [16]), usually the items of existing questionnaires are used.

In the *scale construction* stage, the external method focuses on the strength of the relationship between items and the criterion. Items that show a high correlation with the criterion, but low correlations among them are optimal for prediction (e.g., [73, 82, 83]). Items with a negative relation are usually reversed in the scoring rule.

In the *evaluation* stage, the stability of the item-criterion and scale-criterion relations is studied in a new sample from the target population. As is the case for the internal method, cross-validation is needed to prevent capitalization on chance. In addition, to determine the reliability of the scale, test–retest reliability coefficients are usually obtained [31]. In general, the external method tends to produce scales with low internal consistency coefficients [84], which is not surprising because heterogeneity instead of homogeneity is emphasized. Because the external method focuses on empirical relationships, it cannot be known if the resulting scale performs well on other criteria such as face validity and construct validity.

The external method has been criticized because it tends to result in scales with heterogeneous content, which may therefore lack meaningfulness and interpretability [36, 85]. In addition, it has also been suggested that such scales do not follow a reflective model (in which the construct ‘causes’ the item scores), but a formative model [86–88] (in which the construct is determined by the items), by which they would be inappropriate for measurement purposes (e.g., [62, Chap. 6]).

### The construct method

The construct method [41, 89] is guided by an explicit theory about the construct and uses it to generate hypotheses about the questionnaire which are tested empirically. It is therefore applicable only if sufficient formal knowledge is available. The construct method has a cyclic character: if the items or the scales are found to violate the construct theory, construction is undertaken anew by revising the questionnaire. The method is also known as the ‘substantive’ method [30], the ‘rational’ method [32], and the ‘Jacksonian’ method [90] and has its origin in the standards for test developers and users issued by the American Psychological Association in 1954, which defined a new type of validity, named ‘construct validity’ (e.g., [91, 92]). This type should be distinguished from the more general one used to denote the validity of a test (‘the test measures what it aims to measure,’ e.g., [93]). One of the central claims is that the meaning of a scale cannot be known until it has been empirically embedded in a nomological net, which is a theoretical network of associations of the construct with other variables derived from the construct theory [94]. Examples of questionnaires developed using this method are the Personality Research Form [95] and the Quality of Life in Dementia questionnaire [96].

The *concept analysis* of the construct method is guided by construct theory, often expressed in a nomological network, taking into account important variables, and specifying the assumed relationships among them. An operational definition of the construct at hand is provided, and related and confounding variables are specified (cf. [91, 97]). Related variables are variables that may be correlated to the construct of interest, but are conceptually distinct. For example, when constructing a questionnaire for assessing quality of life in patients with dementia, related variables would be depression and dementia severity [98]. Confounding variables are variables like social desirability, and other response sets, that may bias measurement. Furthermore, different conceptualizations of the domain should be identified and taken into account.

In the *item production* stage, the operational definition is used to generate the items. The related and confounding variables are also taken into account. For example, the kind of judgments the respondents are able to make and what knowledge can be taken for granted are also considered. Furthermore, the constructor pays attention to aspects such as item wording, because items may correlate due to semantic overlap alone. Often, the theoretical relevance, content, and semantic features of the items are judged by experts and potential respondents. Furthermore, a pilot study is often carried out to verify whether the items behave as expected. If necessary, items are rewritten or discarded.

After a first administration of the item set, *scale construction* takes place on the basis of content saturation [41], which refers to the convergent and discriminant validity of the items. Items that correlate highly with the intended scale, and substantially more weakly with scales measuring distinct constructs, are characterized by good content saturation and are retained. Items that show low convergent and discriminant validity are possibly discarded. However, decisions about items are usually not solely made on the basis of item statistics; the origin of poor item functioning is studied as well. It may be that the original conceptualization was flawed, or that the results were confounded in some way. For example, unexpected outcomes may have been the result of an unintentional narrowing of the scale content. If most of the items refer to behavior, the one or two items referring to cognitions may have low correlations with the other items. Under the construct method such results typically lead to a reconsideration of the content of the other items as well.

In the *evaluation* stage, a validation sample is obtained and the nomological network with its presumed relationships is tested empirically. Sometimes a multitrait-multimethod design [99] is used to assess the convergent and discriminant validity of the items and scales [100, 101]. In addition, often confirmatory factor analysis is performed assuming a simple (or ‘between-item’) structure in which each item is linked to a single construct. Other analyses typically performed in this stage are reliability analysis and differential item functioning.

The construct method, as one of the deductive methods, has been recommended because it is claimed that it produces scales with favorable psychometric properties compared to intuitive and inductive methods [102]. However, the role of the nomological net in construct validity has received criticism with reference to its philosophical fundaments by validity theorists (e.g., [73, 103]). In short: Although they may be useful for building and testing construct theory, empirical correlations with other variables would not allow for identifying what a scale actually measures.

### The facet design method

The facet design method [104, 105] is guided by content validity and entails a systematic and comprehensive specification of the construct which ensures that the items in a questionnaire are representative of that construct. It starts with an inventory of the construct domain and divides it into a number of aspects, called facets. Each facet, in turn, consists of facet elements; facets are crossed in order to fully span the construct domain [106]. This design corresponds to the factorial design for experimentation [107]. Like the construct method, the facet design method is a hypothesis testing method, and the assumed structure is tested empirically. By contrast, formal theory about the construct and its relation with other variables does not play a central role in the facet design method. It is particularly suitable if formal knowledge of the construct domain and its facets is available, or can be acquired easily. An example of a questionnaire constructed according to the facet design method is the Dental Anxiety Questionnaire [108]; in addition, Landsheer and Boeije [109] illustrated how to use it to improve the Obesity Cognition Questionnaire.

The *concept analysis*, which forms the core of the facet design, consists of four steps. First, an inventory is made of the behavioral features and underlying processes that are essential to the definition of the construct. Fear, for example, can be viewed as either a physiological reaction, a cognitive process, an affectional state, or a behavioral response, and all these aspects should be represented if an anxiety questionnaire were to be constructed. Second, elaborating on this inventory the facets are defined. Facets should be independent and mutually exclusive aspects of the domain. For example, Stouthard et al. [108] developed a questionnaire for dental anxiety, distinguishing, among other things, a time facet, a reaction facet, and a situation facet. Third, for each facet, its elements are determined, which should be mutually exclusive categories and fully cover the content domain. To illustrate, Stouthard et al. [108] distinguished four elements of the time facet: at home, on the way to the dentist, in the dentist’s waiting room, and in the dental chair. Fourth, the final structure of the facet design is determined by combining the facets. For example, in the questionnaire of Stouthard et al. [108], one of the combinations was the extent to which a patient (a) is afraid (b) at home when (c) she thinks about the dentist performing treatment. Every cell in the facet design defines a manifestation of the construct and the complete facet design is assumed to fully map the construct.

As the cells are defined by their constituent facet elements, at the start of the *item production* stage the required item content is completely known. The total number of items needed depends on the size of the facet design, and the number of required items per cell. Each item is produced by creating content for the combination of the facet elements. After a first round of writing items the result is judged in terms of its coverage of the facet design. If problems are encountered it may be indicative of a flawed facet design, which may lead to a modification of the original facet design.

In the *scale construction* stage, the set of items is investigated using a pilot administration in a sample from the target population. From the facet design, specific hypotheses about the structure underlying the item scores follow [110–113]. For example, it is expected that items that belong to the same cell are more alike than items that belong to different cells. Multidimensional scaling can be used to determine whether the item responses are compatible with the hypothesized structure [114, 115]. Alternatively, using confirmatory factor analysis, the facet design can be represented by a number of factors, e.g., a general factor, and a specific factor for every facet element [107]. Note that these factor models should be distinguished from those used under the internal and construct methods, as they adhere to a complex (or ‘within-item’) structure. In both approaches, items violating the facet structure are identified, and possibly removed from the scale.

The *evaluation* stage does not contain specific procedures to assess the validity of the instrument. Content validity is usually claimed by referring to the full coverage of the construct domain as defined in the concept analysis. Sometimes the assumed item structure is tested in an independent sample to assess the effects of capitalization on chance in the scale construction phase. In addition, the reliability and validity of the questionnaire are usually determined as well.

Like the construct method, the facet method has been recommended since it has been claimed to produce scales with favorable psychometric properties [102]. However, the concept of a content domain, and content validity itself have been topics of debate among validity theorists [62, 116]. For example, it has been claimed that only a content domain for which it is theoretically possible to construct an infinite set of items allows for a reflective interpretation; by contrast, a content domain for which such an infinite set would be impossible is compatible with a formative interpretation [62, Chap. 5].

In addition, it may be claimed that the facet design method is related to the prototypical method in that both methods sample items from a behavior domain. They differ, however, in the sampling plan used: The facet design method is used to fully cover the domain and therefore adheres to stratified sampling; the prototypical method is used to sample typical behaviors by which it adheres to purposive sampling [117].

## Discussion

A new taxonomy of methods for questionnaire design was introduced which links available procedures to a specific test goal. It contains four stages of test construction to describe prototypes of each method: concept analysis, item production, scale construction, and evaluation. The scale construction stage, in which items are selected into a scale, is used for identifying methods. Six methods are distinguished, each related to a specific psychometric aspect relevant for serving a test goal. The purpose of the taxonomy is to provide a clear structure for classifying the multitude of methods for test construction; it has, therefore, a descriptive instead of a normative nature. In other words, no claims are made that the taxonomy be used to specify best practices for test construction.

For a taxonomy to be valid it (a) should have categories that are mutually exclusive, and (b) should be exhaustive, that is, capture all the available elements. The six psychometric aspects used to categorize the methods are evidently mutually exclusive. However, it is recognized that the taxonomy presents prototypes, that specific procedures may vary in practice, and approaches considering several aspects at a time are conceivable. For example, one could generate items with an act nomination procedure, and focus on homogeneity in the scale construction phase. In the taxonomy, such a combination would be classified as an internal method, however, because the scale construction stage is used for identifying methods. The exhaustiveness of the taxonomy was secured by an inclusion of all psychometric aspects deemed important in literature. Implicitly, the claim is made that if a new method would emerge, it coincides with the recognition of a new psychometric aspect.

Due to its teleological nature, the taxonomy connects well to current theories of validity as it links the goals encountered in test construction to procedures used in test validation (i.e., in gathering *evidence* of validity). Some theorists claim that there is only a single concept of validity (‘the test measures what it aims to measure’) and that the different subtypes of validity, such as face validity, construct validity, and so on, are not aspects of it but refer to the different research procedures used for validation [72, 94, 103]. In addition, it is also recognized that each sort of evidence adheres to a specific test goal, which means that when validating a questionnaire not all aspects can receive equal consideration, and that a test should be primarily evaluated using the type of evidence associated with the original goal of the test (cf., [62, p. 302]).

In the taxonomy, the optimization of one aspect implies that other aspects may not be optimized, and therefore that a scale possibly shows deficiencies on aspects that are not central to the test developer. Each of the methods then has a particular strength, but possibly some weaknesses as well. The tradeoff among psychometric aspects is most easily shown for the internal and external methods as for both their central aspect may be quantified. By optimizing homogeneity, utilizing the internal method, instruments tend to show lower criterion validity, and by stressing criterion validity, externally developed instruments tend to show lower homogeneity (for mathematical proofs, and an empirical illustration, see, [84]). Similarly, the rational method produces instruments for which the reliability, content validity, and construct validity are not optimized, and it therefore seems reasonable to assume that they perform relatively poor on these psychometric qualities. Likewise, the prototypical, the internal, the external, and to some extent the facet method result in instruments lacking a theoretical basis and may therefore show deficiencies regarding construct validity.

The previous discussions might lead the reader to wonder if the taxonomy is an invitation to pick one psychometric aspect to the exclusion of others. The answer is no. Developing a questionnaire to optimize a single aspect is expected to result in a questionnaire of little use as it is rather unlikely that it meets minimal requirements for other aspects. Rather, the taxonomy is intended to raise awareness about potential priorities and trade-offs in test construction. Moreover, in a world of limited resources, test constructors cannot be expected to provide a full mapping of all aspects of a questionnaire. The taxonomy may help to set up a test construction plan in which the priorities among the psychometric aspects are made explicit.

In the third section, the taxonomy was illustrated using prototypical examples for each category, but it is important to acknowledge that in practice, test construction often consists of a mixture of methods and that across the stages and studies involved in the development of a questionnaire the focus often shifts. Again, the taxonomy may help to conceptualize these shifts in focus more clearly. A research team could start the development of a new questionnaire for measuring insomnia with a literature search in the concept analysis stage to obtain items from previous research on the assessment of insomnia. In the item production stage, the researchers could further draw on the knowledge of (a) experts from the research field and (b) patients with sleeping problems to select, adjust, and possibly extend the item set from the first stage (which is typical for the rational method). In the scale construction stage, they could plan a first evaluation of the developed items in a large sample from the target population to assess the degree to which the items are interrelated (which is typical for the internal method). It is in this stage that the priority switches from face validity to homogeneity and it is conceivable that removing items that do not meet homogeneity requirements has negative consequences for face validity, which was the focus of the previous stages. Similarly, in the evaluation stage, focus switches to other aspects such as criterion and content validity, and it is uncertain how well the set of remaining items performs on these aspects as they received no priority in the previous stages. This example shows the link with all other design activities: the process of creating, adjusting, and selecting items is guided by the focus on one or more product features. When a psychometric aspect is not given priority, the final item set may not perform well on it. Moreover, if two aspects have a tradeoff, giving priority to the one aspect may lead to an item set that does worse on the other.

In the second section, it was shown that the six methods could be further classified into three broad classes of two methods each: the intuitive, inductive, and deductive methods. This tripartite arrangement can also be used to link the state of knowledge about a construct to the usefulness of methods for questionnaire construction. An intuitive method (rational or prototypical) seems useful when the designer only has informal knowledge of the construct. An inductive method (internal or external) is useful when there is a global knowledge from prior research about the construct, including one or more provisional instruments. A deductive method (construct or facet design) would be useful only if considerable knowledge from previous research about the content and structure of the construct is available. The argument may also be reversed: The prevalence of methods of questionnaire design in a research field is indicative of the amount of knowledge available about the constructs that are central to it. For example, since in the field of quality of life research the rational and internal methods are most frequently used, one might conclude that there still is a lot to be gained in theory development.

## Footnotes

1 Some scholars (e.g., [62, Chap. 6]) have warned against using principal components analysis for modeling questionnaire data as it would amount to a formative model, which does not allow for testing the hypothesis that the item responses are induced by an underlying common factor.
